# The importance of the perioperative nurse

**Published:** 2020-12-31

**Authors:** Ciku Mathenge

**Affiliations:** 1 Professor of Ophthalmology: University of Rwanda and Director of Training and Research: Rwanda International Institute of Ophthalmology, Rwanda.

**Qualified nurses play an important role in the operation room before, during and after surgery.**

The perioperative nurse is an essential member of the team when operating on patients with eye-related conditions. Indeed, whenever I'm required to operate in remote locations, my core request - apart from a microscope and surgical instruments - is that I travel with a dedicated perioperative nurse.

Surgeons are the most visible members of the cluster of gowned figures gathered around operating tables all around the world. However, the surgeon is always a part of a surgical team and is supported by a number of other highly trained professionals, each with a clearly defined role and serving a vital function. In many parts of the world, qualified nurses play multiple roles in the operating room.

As an eye surgeon, I consider the nurse to be one of the most important people in the perioperative space - before, during, and after surgery. These nurses are known by various names in different places: scrub nurses, operating room (OR) nurses, circulating nurses, surgical technicians, theatre nurses/assistants, or operating room technicians. I will use the term perioperative nurse to encompass all these roles.

**Figure:**
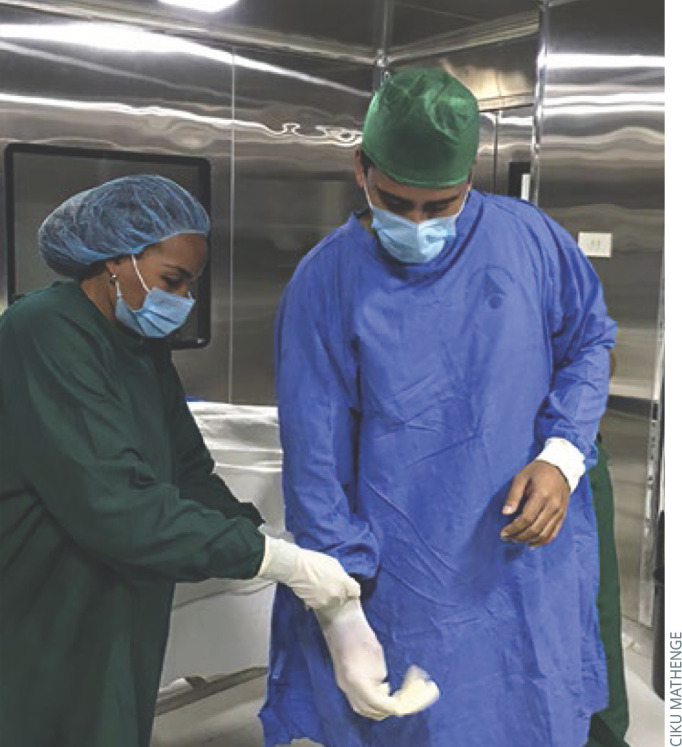
The perioperative nurse helps an ophthalmologists to don his gloves. **RWANDA**

The key responsibility for the perioperative nurse is to maintain a sterile environment for the patient and surgical team before, during, and after surgery. Consequently, the nurse often has multiple responsibilities, especially where there are shortages of skilled health workers.

## Before surgery

Before surgery, the perioperative nurse may be responsible for supervising the transport of patients to and from the theatre and wards. The nurse will also prepare the patient for the surgical procedure, i.e.:

- Checking the patient's records
- Checking vital signs
- Washing, shaving, and disinfecting surgical sites
- Ensuring that the correct eye is labelled.

Perioperative nurses arrive before the procedure to set up the room and check its condition in order to ensure a clean, safe and efficient environment for patients and surgeons. They ensure that adequate supplies are available and check for correct positioning: making sure the patient is well positioned on the table, that the surgeon's stool is adjusted to the correct height, and that the microscope and all of its viewing stems are working well. They need to be familiar with the correct operation of all equipment in the operating room, including phacoemulsification machines, vitrectomy machines, and lasers, if relevant. Perioperative nurses are also responsible for collecting, checking, and returning the equipment needed for each procedure. For example, before a retinal detachment operation starts, they must make sure that the cryotherapy cylinder contains enough liquid nitrogen, even though the surgeon may not use it. Similarly, during a cataract operation, they must check that all the correct lenses and viscoelastic options are available, just in case they are needed. Often, the perioperative nurse has to not only anticipate numerous complications that could be encountered during the procedure, but also the needs of multiple surgeons with different individual preferences and levels of skill.

## During surgery

In the scrub role, a perioperative nurse will ensure all the gloves needed are available in the correct sizes. She or he will be the first to scrub in for a procedure and assist the remaining team members with gowning and gloving. During the operation, the perioperative nurse will hand the surgeon any necessary instruments, sponges, and other items, and provide retraction, suction, or irrigation of the eye as directed. In this role, the perioperative nurse requires a deep enough knowledge of the procedure to anticipate the surgeon's needs and have the correct items ready to hand over. This skill allows the surgeon to not break concentration and manage any complications optimally. For example, as soon as vitreous presents during a cataract procedure, the perioperative nurse prepares for vitrectomy, even before the surgeon asks. Often, experienced nurses will offer invaluable advice to novice surgeons such as, “Perhaps you should increase the incision size?” Such advice is always appreciated and emphasises the key role of the perioperative nurse within the operating theatre.

Within the operating room, the perioperative nurse may also work outside the sterile field (sometimes called the circulating nurse). Circulating nurses provide additional supplies and sterile instruments as needed during the operation and assist the other team members in monitoring the status of the patient or helping with the repositioning of the patient during the procedure.

**Figure:**
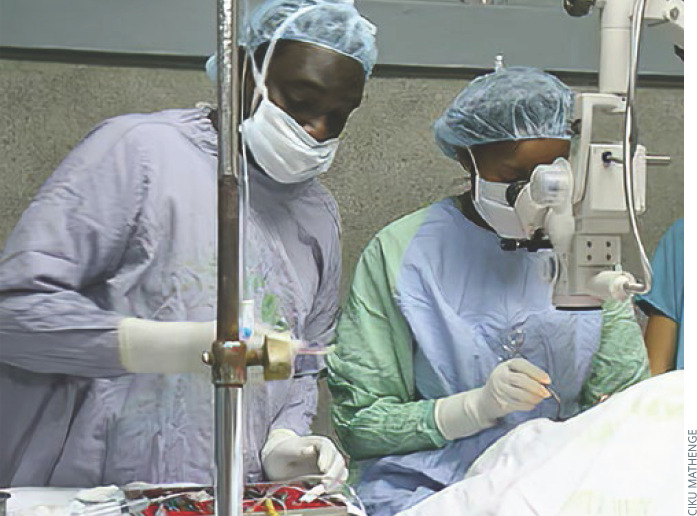
The perioperative nurse assists during eye surgery. **RWANDA**

## After surgery

After the operation, the surgeon often writes her or his notes and leaves the room. The perioperative nurse may then be responsible for monitoring the patient's condition and remaining alert for any indicators revealing a good or bad outcome. The nurse will often be responsible for giving the correct postoperative instructions to patients before they go home - something that can greatly impact outcomes.

**“The nurse will often be responsible for giving the correct postoperative instructions to patients before they go home - something that can greatly impact outcomes.”**

In cases where general anaesthesia was used, such as in paediatric ophthalmology, nurses will continually evaluate the patients until they wake up and help them understand where they are and what is going on as they awaken from the anaesthesia. Other nursing interventions will include monitoring vital signs, airway patency, and neurologic status; managing pain; assessing the surgical site; assessing and maintaining fluid and electrolyte balance; and providing a thorough report of the patient's status to the surgeon and the patient's family.

## Other duties

Many of the nurses who work in the operating room tend to be senior, very experienced nurses. As a result, they might also spend part of their time on training, supervisory or administrative duties.

As surgeons, we are sometimes unaware of all that goes on in order to make the surgical experience smooth for the patient and surgeon. Much of this rests in the hands of the perioperative nurse - individuals who not only work with precision but also have the ability to think on their feet, act on core scientific principles, adapt to ever-changing circumstances and take the initiative to do what is necessary and right in each surgical situation.

